# Smoking-associated upregulation of CBX3 suppresses ARHGAP24 expression to activate Rac1 signaling and promote tumor progression in lung adenocarcinoma

**DOI:** 10.1038/s41388-021-02114-8

**Published:** 2021-11-16

**Authors:** Xin Jin, Bin Zhang, Hao Zhang, Haixin Yu

**Affiliations:** 1 Department of Urology, The Second Xiangya Hospital, Central South University, Changsha, Hunan 410011 China; 2 Uro-Oncology Institute of Central South University, Changsha, Hunan 410011 China; 3 Cancer center, Union Hospital, Tongji Medical College, Huazhong University of Science and Technology, Wuhan, 430022 China; 4 Department of Cardiovascular Surgery, The Second Xiangya Hospital, Central South University, Changsha, Hunan 410011 China

**Keywords:** Non-small-cell lung cancer, Oncogenes

## Abstract

Although tobacco smoking is a risk factor for lung adenocarcinoma (LUAD), the mechanisms by which tobacco smoking induces LUAD development remain elusive. Histone methylation levels in human bronchial epithelial cells have been reported to increase after exposure to cigarettes. In this study, we explored the mechanisms regulating histone methylation in LUAD in response to smoking. We found that the histone H3K9 methylation reader CBX3 was upregulated in current smokers with LUAD, and that CBX3 overexpression promoted LUAD progression. Functional enrichment analyses revealed that CBX3 regulated the activation of Rho GTPases in LUAD. We also found that by forming a complex with TRIM28, TRIM24, and RBBP4, CBX3 repressed the expression of *ARHGAP24* and increased the amount of active Rac1 in LUAD cells. Collectively, these results suggest that smoking associated upregulation of CBX3 promotes LUAD progression by activating the ARHGAP24/Rac1 pathway. Hence, the CBX3/ARHGAP24/Rac1 axis may represent a promising therapeutic target in smoking-induced LUAD.

## Introduction

Lung cancer is one of the most common malignancies and the leading cause of cancer-related death worldwide, with approximately 2,207,000 new cases and 1,796,000 deaths in 2020 [1]. Lung adenocarcinoma (LUAD) is the most common histological subtype of lung cancer, accounting for approximately 40% of lung cancer cases. The incidence of LUAD has significantly increased in recent years and continues to rise globally. Tobacco smoking has been identified as a strong risk factor for lung cancer. Smoking has been shown to affect the epigenetic modification of genes in lung tissues and the blood [2, 3]. In addition, exposure to cigarettes has been linked with increased levels of whole-genome histone methylation in human bronchial epithelial cells; this was particularly evident for mono- and dimethylation of histone H3 and H4 residues [4]. The expression of histone methyltransferase (HMT), an enzyme catalyzing histone methylation, has also been found to be regulated by smoking [5, 6]. Therefore, understanding the role of smoking in histone methylation and transcriptional regulation may facilitate the development of novel therapies for smoking-induced LUAD.

Chromobox homolog 3 (CBX3) belonging to the heterochromatin-associated protein 1 (HP1) family, binds to chromatin and trimethyl lysine 9 of histone H3 (H3K9me3) to promote the formation of heterochromatin [7]. By suppressing the expression of various genes, CBX3 regulates numerous cellular processes, including cell growth, cell differentiation, and DNA damage [8–10]. Aberrant CBX3 expression has been implicated in the development of various cancer types [11]. However, the role of CBX3 in LUAD remains understudied [12]. In addition, little is known about the relationship between smoking, CBX3, and LUAD progression.

Here, through in silico analyses of publicly available datasets of genes involved in histone methylation, we found that the expression of the histone H3K9 methylation reader CBX3 was upregulated in current smokers with LUAD. We also found that CBX3 overexpression was associated with LUAD progression. Functional enrichment analyses revealed that CBX3 regulates numerous signaling pathways, including Rho GTPase signaling. We also found that CBX3 represses the expression of ARHGAP24, a negative regulator of the Rho GTPase pathway [13], thereby increasing the amount of active Rac1 in LUAD cells. Collectively, these data unveiled a previously unknown relationship between smoking, CBX3, and Rac1 in LUAD.

## Material and methods

### Data mining and bioinformatics analysis

The Cancer Genome Atlas (TCGA), Gene Expression Omnibus (GEO), Clinical Proteomic Tumor Analysis Consortium (CPTAC), CancerSEA, and ChIP-Atlas were used for data mining and bioinformatics analysis (see Supplementary Methods for details).

### Cell lines

Lung adenocarcinoma cell lines including A549 (CM-0016) and H1299 (CM-0165) were purchased from Procell (Wuhan, China). Both cell lines were authenticated by short tandem repeat (STR) profiling. A549 cells were cultured in Ham’s F-12K medium (PM150910, Procell) supplemented with 10% FBS (164210-500, Procell) and 1% P/S (PB180120, Procell). H1299 cells were cultured in RPMI-1640 medium (PM150110, Procell) plus 10% FBS (164210-500, Procell) and 1% P/S(PB180120, Procell). All cell lines were routinely maintained at 37 °C in a 5% CO_2_ incubator.

### Western blot analysis and coimmunoprecipitation (Co-IP)

Cells were harvested and lysed with lysis buffer containing 1% protease and phosphatase inhibitors for 15 min on ice as described previously [14, 15]. A protein assay kit (Pierce Biotechnology, USA) was used to determine the protein concentration. Equal amounts of proteins were separated in SDS-PAGE gels and detected in PVDF membranes after incubation with primary antibodies and subsequent secondary antibodies. Antibodies against the following proteins were used: CBX3 (cat. no. 11650-2-AP, Proteintech; 1:1000 dilution); ARHGAP24 (cat. no. 18834-1-AP, Proteintech; 1:2500 dilution); Rac1 (cat. no. 66122-1-Ig, Proteintech; 1:2500 dilution); GAPDH (cat. no. 60004-1-Ig, Proteintech; 1:5000 dilution); TRIM24 (cat. no. 14208-1-AP. Proteintech; 1:1000 dilution), TRIM28 (cat. no. 15202-1-AP; Proteintech; 1:2000 dilution), H3K9me3 (cat. no. ab8898; Abcam; 1:1000 dilution) and RBBP4 (cat. no. ab79416; Abcam; 1:1000 dilution).

For immunoprecipitation, the cell lysate was incubated with Pierce Protein G Agarose (Thermo Fisher Scientific, USA) and primary antibody or IgG. Then the precipitates were analyzed by immunoblotting. To detect active Rac1, we followed the manufacturer’s instruction for an Active Rac1 Detection Kit (cat. no. 8815, Cell Signaling Technology).

### Quantitative real-time PCR (RT-qPCR), chromatin immunoprecipitation (ChIP) and ChIP-qPCR

The RT-qPCR procedure was described previously. A PrimeScript™ RT reagent kit (cat. no. RR037A) and TB Green™ Fast qPCR Mix kit (cat. no. RR430A) purchased from Takara Bio Inc. (Shiga, Japan) were used to construct cDNA and perform RT-qPCR analysis. The sequences of primers are provided in Table S1.

A chromatin Extraction Kit (Abcam, ab117152, USA) and ChIP Kit Magnetic - One Step (Abcam, ab156907, USA) were used to preform ChIP as described previously. The detailed antibodies as follows: CBX3 (Proteintech; 11650-2-AP; 1:100), TRIM24 (Proteintech; 14208-1-AP; 1:500), TRIM28 (Proteintech; 15202-1-AP; 1:200), H3K9me3 ((Abcam; ab8898; 1:500) and RBBP4 (Abcam; ab79416; 1:100). The sequences of primers are provided in Table S2.

### Tissue microarray and immunohistochemistry (IHC)

A tissue microarray (cat. no. D881001, Bioaitech, CN) and IHC were employed to assess the levels of CBX3 (cat. no. 11650-2-AP, Proteintech; 1:2500 dilution) and ARHGAP24 (cat. no. 18834-1-AP, Proteintech; 1:2500 dilution) in lung adenocarcinoma. The IHC score was evaluated as previously reported.

### Xenografts assay

Ethical approval was obtained from the Ethics Committee of Tongji Medical College, Huazhong University of Science and Technology for all animal procedures. BALB/c-nude mice (4–5 weeks old, 18–20 g) were obtained from Vitalriver (Beijing, China). Power analysis was used to calculate the sample size required for animal experiments and animals were randomized into the different groups. A459 cells were transduced with different lentiviral particles. After puromycin selection for 72 h, cells (1 × 10^7^ per mouse) were subcutaneously injected into the backs of mice. The xenografts assay procedure was described previously [16]. At the study endpoint, the volume and mass of xenografts were measured. Nicotine (cat. no. MED24104) was purchased from Medbio (CN).

### Statistical analysis

All data are presented as the means ± standard deviation (SD). The sample size (*n*) for each statistical analysis is provided in the figure legends. Statistical significance was determined with Student’s *t* test, and one-way or two-way ANOVA using GraphPad Prism 5 software. *P* < 0.05 were considered statistically significant.

Other methods, the sequenced of gene-specific shRNAs (Table S3), and demographic information of the clinical data (Table S4, S5) are provided in the Supplementary Information.

## Results

### CBX3 is upregulated in current smokers with LUAD, and CBX3 overexpression predicts worse survival in patients with LUAD

**Fig. 1:**
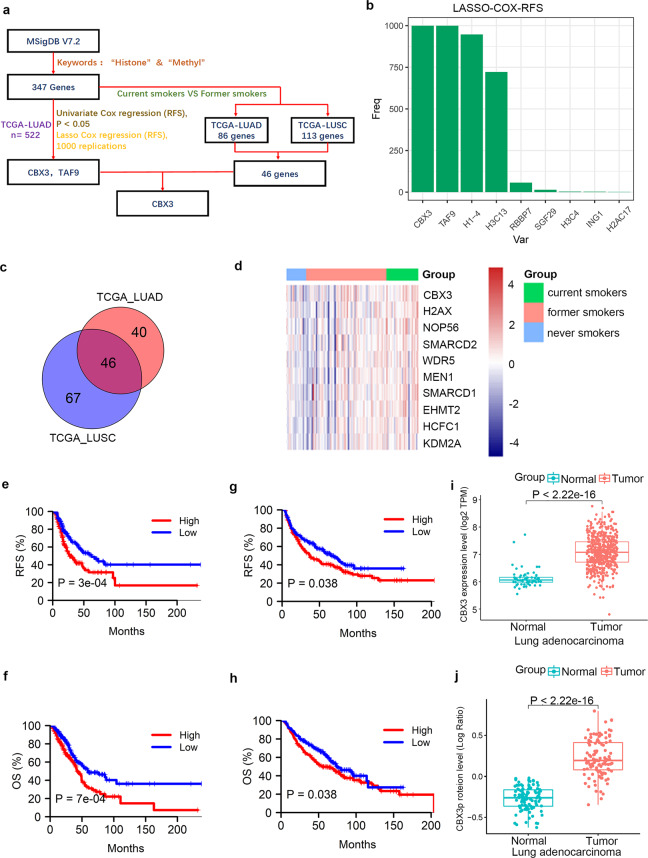
CBX3 is upregulated in current smokers with LUAD, and CBX3 overexpression predicts worse survival in patients with LUAD. **a** The flow chart of how to identify CBX3 associated with survival and smoking in LUAD. **b** 10-fold cross-validation with 1000 replications for selection of genes associated with LUAD RFS in the LASSO-COX model by minimum criteria (the 1-SE criteria). **c** Venn diagrams showing numbers of upregulated genes involved in the histone methylation in current smokers compared to former smokers in TCGA-LUAD and TCGA-LUSC datasets. **d** Heatmap of top 10 averagely highly expressed genes in TCGA-LUAD samples out of the 46 genes showed in Venn diagram. Red indicates overexpression and blue underexpression. The columns correspond to the LUAD samples. The real status of the samples is given in a color scale: blue for never smokers, red for former smokers and green for current smokers. **e**, **f** Kaplan–Meier analysis with two-sided log-rank test was conducted to evaluate the differences in RFS (**e**) and OS (**f**) between the patients with high and low expression of CBX3 in TCGA-LUAD dataset. Median expression level of CBX3 was used as cutoff. **g**, **h** Kaplan–Meier analysis with two-sided log-rank test was conducted to evaluate the differences in RFS (**g**) and OS (**h**) between the patients with high and low expression of CBX3 in GSE68465 dataset. Median expression level of CBX3 was used as cutoff. **i**, **j** Differential expression analyses of CBX3 between tumor and normal tissues in TCGA-LUAD (**i**) and CPTAC-LUAD (**j**) datasets.

Genes (*n* = 347) involved in histone methylation-related biological processes and signaling pathways were identified from the Molecular Signatures Database (MSigDB) (Fig. 1a). Through univariate Cox regression analysis, 22 genes were identified to be associated with the recurrence-free survival (RFS) of LUAD patients in the TCGA-LUAD dataset (*P* < 0.05). Lasso-Cox regression analysis with 1000 replications further showed that *CBX3* and *TAF9* were two key genes (both 1000 times) associated with RFS in patients with LUAD (Fig. 1b). Then, we combined the transcriptome data of the TCGA-LUAD and TCGA-LUSC datasets, and found that *CBX3* but not *TAF9* was expressed at significantly higher levels in the current smoking group than in the former smoking group in both datasets (all *P* < 0.05; Fig. 1c). The low expression level of *CBX3* was mainly concentrated in former smokers and never smokers in the TCGA-LUAD dataset (Fig. 1d). Kaplan–Meier survival analyses showed that high *CBX3* expression levels were associated with poor RFS and overall survival (OS) in both the TCGA-LUAD and GSE68465 datasets (all *P* < 0.05; Fig. 1e–h). Additionally, *CBX3* was significantly upregulated in tumor tissues compared with tumor-adjacent tissues in the TCGA-LUAD and CPTAC-LUAD datasets (all *P* < 0.001, Fig. 1i, j). Similarly, through IHC staining, we detected CBX3 expression in a tissue microarray of lung adenocarcinoma specimens (nontumor lung tissues (*n* = 8) and lung adenocarcinoma tissues (*n* = 59), cat. no. R881001, Bioaitech, China). The IHC images stained with CBX3 are shown in Supplementary figure 1a. We found that CBX3 was downregulated in the nontumor lung tissue compared to the lung adenocarcinoma (*P* = 0.0031) (Supplementary Fig. 1a, b and Table S5).

### CBX3 promotes LUAD cell growth and invasion

**Fig. 2:**
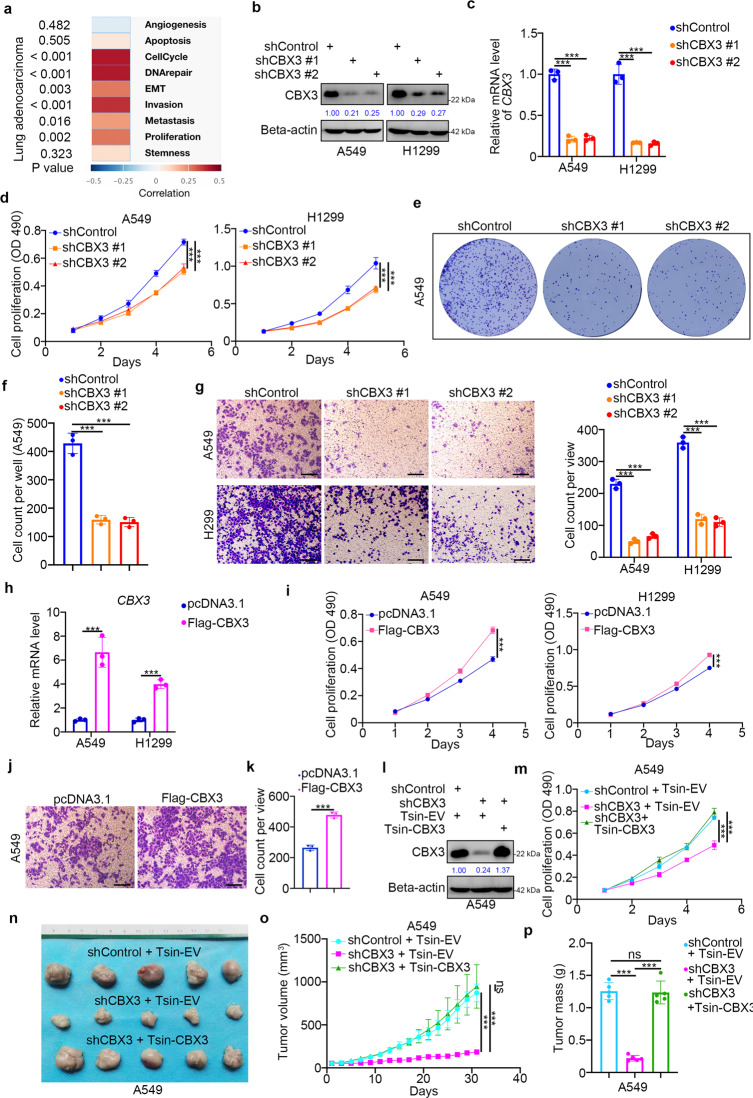
CBX3 promotes LUAD cell growth and invasion. **a** Analysis the potential cancer-related function of CBX3 in lung adenocarcinoma by the single cell sequencing dataset. **b**–**g** A549 and H1299 cells were infected with shControl, shCBX3 #1, or shCBX3 #2. Seventy two hours post infection, cells were collected for Western blotting analysis (**b**), RT-qPCR analysis (**c**), MTS assay (**d**), colony formation assay (**e** and **f**) and transwell assay (**g**). Statistical significance was determined by one-way ANOVA followed by Tukey’s multiple comparisons test. Data presented as Mean ± SD with three replicates (*n* = 3). ***P < 0.001. **h**–**k** A549 and H1299 were transfected with pcDNA3.1 (as empty vector) or Flag-CBX3 for 24 h. Cells were collected for RT-qPCR analysis (**h**), MTS assay (**i**) and transwell assay (**j**, **k**). Statistical significance was determined by two-side student *t* test. Data presented as Mean ± SD with three replicates (*n* = 3). ***P < 0.001. **l**, **m** A549 cells were infected with Tsin-EV + shControl, Tsin-EV + shCBX3, or Tsin-CBX3 + shCBX3 for 72 h. Cells were harvested for Western blotting analysis (**l**) and MTS assay (**m**). Statistical significance was determined by one-way ANOVA followed by Tukey’s multiple comparisons test. Data presented as Mean ± SD with three replicates (*n* = 3). ***P < 0.001. **n**–**p** A549 cells were infected with Tsin-EV + shControl, Tsin-EV + shCBX3, or Tsin-CBX3 + shCBX3 as indicated. After 72 h puromycine selection, cells were harvested and subcutaneously injected into nude mice for xenografts assay. The image of tumor was shown in panel (**n**). The tumor growth curve was indicated in panel (**o**). The tumor mass was demonstrated in panel (**p**). Statistical significance was determined by one-way ANOVA followed by Tukey’s multiple comparisons test. Data presented as Mean ± SD with five replicates. NS not significant; ***P < 0.001.

Since the single-cell sequence of LUAD indicated that CBX3 may regulate cell cycle, proliferation, invasion, and metastasis (Fig. 2a and Supplementary Fig. 2a), we further explored the protumorigenic role of CBX3 in LUAD cells. To this end, we silenced *CBX3* expression in A549 and H1299 cells using two different short-hairpin RNAs (shRNAs) (Fig. 2b, c). MTS assays and colony formation assays showed that *CBX3* silencing decreased the proliferation ability of LUAD cells (Fig. 2d–f). Similarly, CBX3 knockdown suppressed cell invasion in A549 and H1299 cells (Fig. 2g). Conversely, CBX3 overexpression enhanced cell proliferation and invasion in LUAD cells (Fig. 2h–k, Supplementary Fig. 2b). Moreover, rescuing CBX3 expression in A549 cells with *CBX3* knockdown reinforced the proliferation of cells in vitro and in vivo (Fig. 2l–p, Supplementary Fig. 2c). Together, these data suggest that CBX3 acts as an oncoprotein in LUAD. In addition, we found that there is a close relationship between CBX3 and smoking in LUAD. Next, we investigated the role of CBX3 in smoking associated LUAD. A549 cells were infected with shControl or shCBX3 for 72 h. After puromycin selection, the cells were subcutaneously injected into nude mice. The silencing effect of CBX3 was evaluated via Western blotting and RT-qPCR analysis (Supplementary Fig. 3a, b). Then, each group of mice was randomly divided into three subgroups that were treated with or without cigarette smoke extract (CSE) (0.3 ml/20 g, i.p.) or nicotine (0.5 mg/kg, i.p.) (Supplementary Fig. 3c) [17]. We demonstrated that CSE and nicotine treatment promoted tumor growth in the shControl group (Supplementary Fig. 3d–f). However, knockdown of CBX3 diminished the tumor growth-promoting effect induced by CSE or nicotine treatment in vivo (Supplementary Fig. 3d–f). Furthermore, knockdown of CBX3 by infection with shRNAs or treatment with CSE or nicotine had no effect on the liver and renal function of nude mice according to serum analysis of aminoleucine transferase (ALT), aspartate transaminase (AST), creatinine (CRE), or blood urea nitrogen (BUN) in each group of nude mice (Supplementary Fig. 3g–j). Together, these data suggest that CBX3 plays a role in modulating smoking-associated lung adenocarcinoma cell growth.

### CBX3 increases the amount of active Rac1 in LUAD

**Fig. 3:**
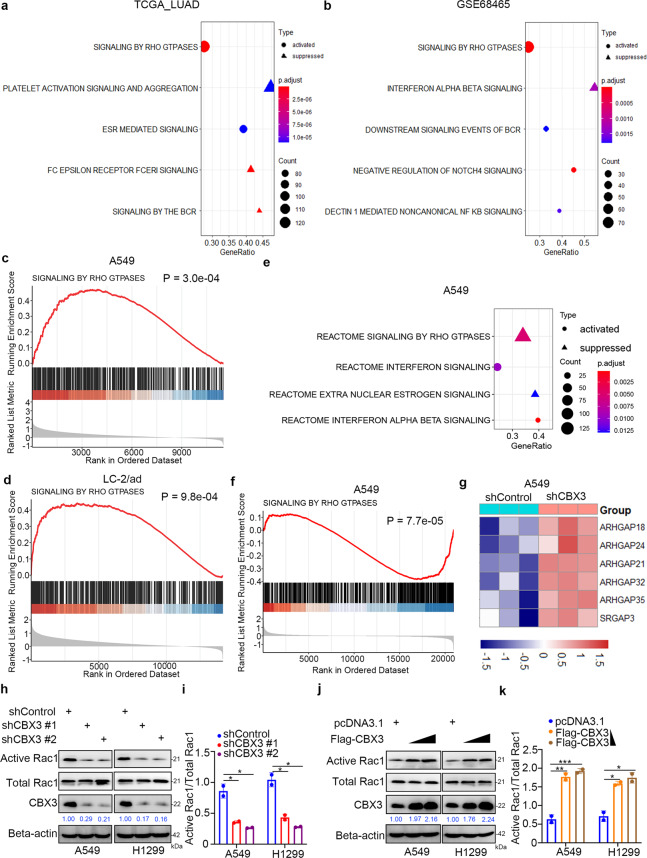
CBX3 increases the amount of active Rac1 in LUAD. **a**, **b** GSEA for CBX3 in TCGA-LUAD (**a**) and GSE68465 (**b**) datasets. Samples were first divided into two groups according to the median expression level of the *CBX3*. Then, differential expression analysis was applied between the high and low expression groups. Input genes for GSEA were sorted by their logFC values. Signaling pathways activated or suppressed by *CBX3* were decided by the NES value derived from GSEA. **c**, **d** Rho GTPase signaling pathway was activated by the over expression of CBX3 in single cells of A459 (**c**) and LC-2/ad (**d**). Median expression level of *CBX3* was used as cutoff. **e**, **f** Knockdown of CBX3 in A549 cell line leaded to the suppression of Rho GTPases signaling pathway. **g** Key genes involved in the regulation of Rho GTPases signaling pathway by the knockdown of CBX3 in A459 cell line. **h**, **i** A459 and H1299 cells were infected with shConrtol or shCBX3 for 72 h. Cells were divided in to two equal parts. The first part of cells was collected for Western blotting analysis and detected by the CBX3, Rac1 and Beta-actin antibodies. The second part of cells were lysed and subjected to GST-pull down following the protocol of Active Rac1 Detection Kit, the active Rac1 was detected by Western blotting analysis. Statistical significance was determined by one-way ANOVA followed by Tukey’s multiple comparisons test. Data presented as Mean ± SD with two replicates (*n* = 2). *P < 0.05. **j**, **k** A459 and H1299 cells were transfected with indicated constructs for 24 h. Cells were divided in to two equal parts. The first part of cells was collected for Western blotting analysis and detected by the CBX3, Rac1 and Beta-actin antibodies. The second part of cells were lysed and subjected to GST-pull down following the protocol of Active Rac1 Detection Kit, the active Rac1 was detected by Western blotting analysis. Statistical significance was determined by one-way ANOVA followed by Tukey’s multiple comparisons test. Data presented as Mean ± SD with two replicates (*n* = 2). *P < 0.05; **P < 0.01; ***P < 0.001.

To explore the mechanisms underlying the oncogenic effects of CBX3 in LUAD, we performed functional enrichment analyses of transcriptomics data of bulk tissues and single cells. Gene set enrichment analysis (GSEA) showed that CBX3 activated Rho GTPase signaling in bulk tissues (TCGA-LUAD and GSE68465) and A459 and LC-2/ad single cells (all *P* < 0.001; Fig. 3a–d). RNA-seq analysis of A549 cells after CBX3 knockdown (GSE173858) confirmed a role of CBX3 in the activation of Rho GTPase signaling (Fig. 3e, f) and indicated that CBX3 downregulated several negative regulators of Rho GTPase signaling in A549 cells (Fig. 3g). Consistently, CBX3 silencing decreased the levels of activated Rac1 in LUAD cells (Fig. 3h, i); Rac1 is considered an indicator of Rho GTPase signaling activation [18]. Conversely, CBX3 overexpression increased Rac1 levels in A549 and H1299 cells (Fig. 3j, k). Taken together, these data suggest that CBX3 plays a critical role in upregulating the amount of active Rac1 in LUAD cells.

### CBX3 negatively regulates *ARHGAP24* expression in LUAD

**Fig. 4:**
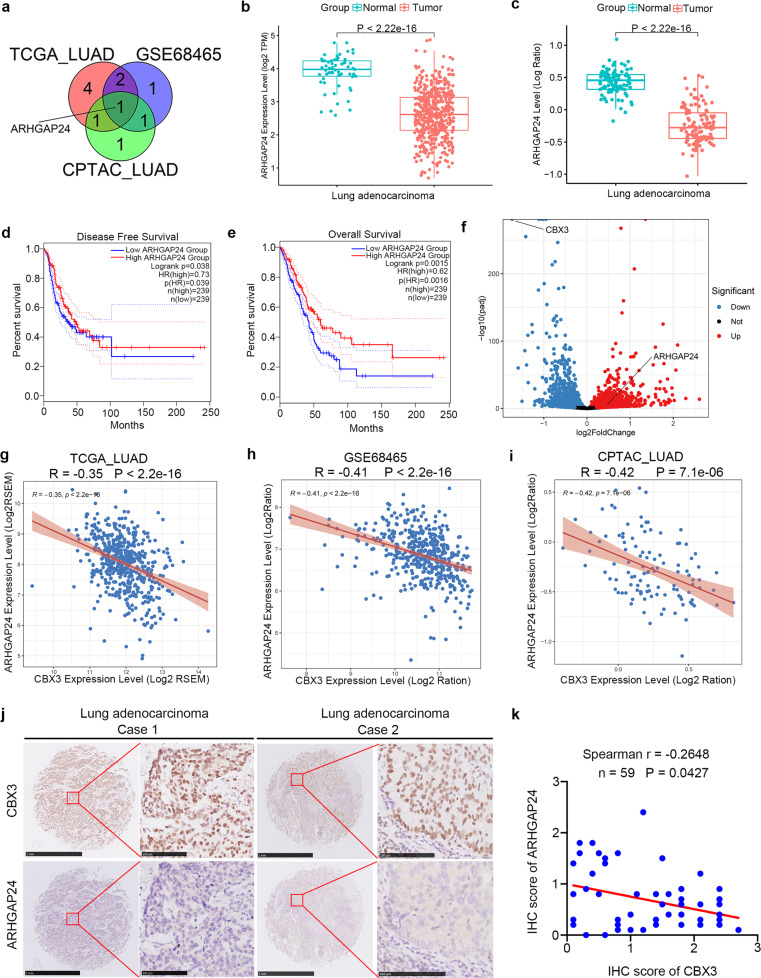
CBX3 negatively regulates ARHGAP24 expression in LUAD. **a** Venn diagram showed number of genes negatively correlated with CBX3 in TCGA-LUAD, GSE68465 and CPTAC-LUAD datasets. **b**, **c** Differential expression analyses of ARHGAP24 between tumor and normal tissues in TCGA-LUAD (**b**) and CPTAC-LUAD (**c**) datasets. **d**, **e** Kaplan–Meier analysis with two-sided log-rank test was conducted to evaluate the differences in RFS (**d**) and OS (**e**) between the patients with high and low expression of ARHGAP24 in TCGA-LUAD dataset. The dashed lines in panel (**d**) and (**e**) represented the 95% confidence intervals of the KM curves. **f** Volcano diagram showed differential expressed genes caused by the knockdown of CBX3 in A549 cell line. **g**–**i** ARHGAP24 was negatively correlated with CBX3 in TCGA-LUAD (**g**), GSE68465 (**h**) and CPTAC-LUAD (**i**) datasets. **j**, **k** The tissue microarray of lung adenocarcinoma was stained with CBX3 and ARHGAP24, respectively (*n* = 59). The typical IHC images stained with CBX3 and ARHGAP24 were shown in panel (**j**). The size of the scale bar on microscopy images as indicated in the figure. The correlation of these two proteins was shown in panel (**k**). Spearman correlation was used to determine statistical significance, *P* = 0.0427.

Next, we explored the molecular mechanisms by which CBX3 regulates the Rac1 pathway. We evaluated the correlation between the levels of CBX3 and regulators of Rho GTPase signaling. ARHGAP24 was the only Rho GTPase regulator negatively associated with CBX3 levels in the TCGA-LUAD, GSE68465, and CPTAC-LUAD datasets (Fig. 4a). In addition, ARHGAP24 mRNA and protein levels were lower in LUAD specimens than in nonmalignant tissues (TCGA-LUAD dataset and CPTAC-LUAD dataset) (Fig. 4b, c). Interestingly, low *ARHGAP24* mRNA levels were associated with poor disease-free survival and OS in patients with LUAD (Fig. 4d, e).

RNA-seq analysis indicated that ARHGAP24 was upregulated in A549 cells after CBX3 silencing (Fig. 4f). Moreover, *CBX3* expression levels were negatively correlated with those of *ARHGAP24* in different LUAD datasets (TCGA, GSE68465 and CPTAC; all *P* < 0.001; Fig. 4g–i). A microarray of LUAD tissues (including tissues of 59 patients with LUAD; cat. no. R881001, Bioaitech, China) was employed to evaluate the protein levels of CBX3 and ARHGAP24 (Fig. 4j). In line with the transcriptomics data, CBX3 protein levels were negatively correlated with ARHGAP24 protein levels in LUAD tissues (Spearman r = −0.2648, *P* = 0.0427; Fig. 4k). These results suggest that CBX3 negatively regulates ARHGAP24 expression in LUAD.

### CBX3 indirectly increases the amount of active Rac1 by repressing ARHGAP24 expression in LUAD cells

**Fig. 5:**
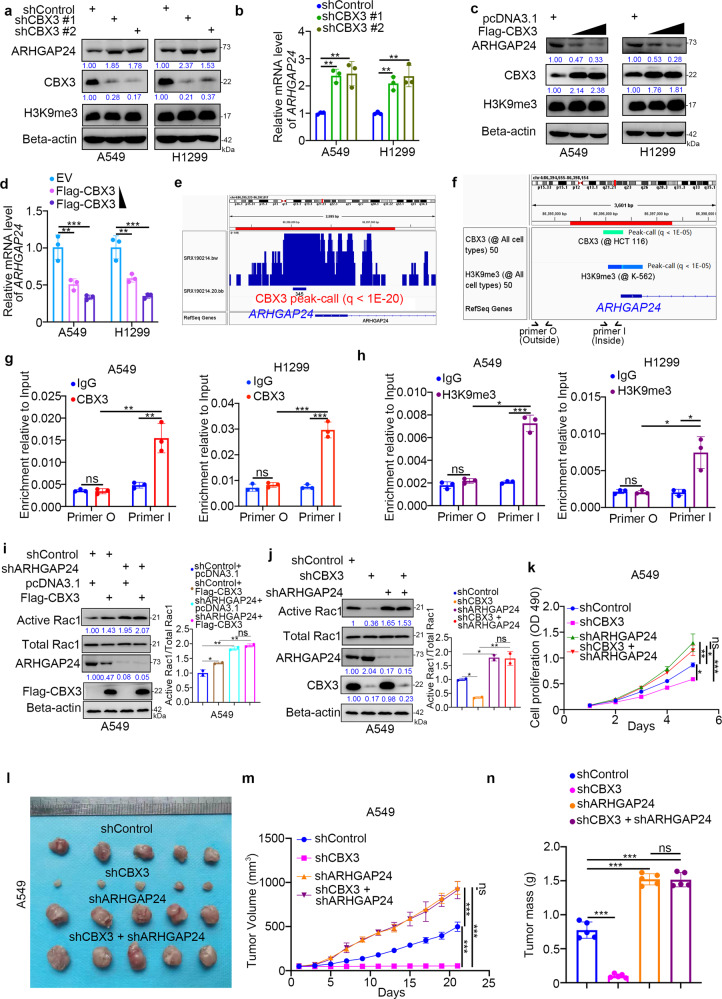
CBX3 indirectly increases the amount of active Rac1 by repressing ARHGAP24 expression in LUAD cells. **a**, **b** A549 and H1299 cells were infected with shControl, shCBX3 #1, or shCBX3 #2 for 72 h. Cells were collected for Western blotting analysis (**a**) and RT-qPCR analysis (**b**). Statistical significance was determined by one-way ANOVA followed by Tukey’s multiple comparisons test. Data presented as Mean ± SD with three replicates. **P < 0.01. **c**, **d** A549 and H1299 cells were transfected with indicated plasmids for 24 h. Cells were collected for Western blotting analysis (**c**) and RT-qPCR analysis (**d**). Statistical significance was determined by one-way ANOVA followed by Tukey’s multiple comparisons test. Data presented as Mean ± SD with three replicates. **P < 0.01; ***P < 0.001. **e**, **f** The ChIP-seq of CBX3 on the promoter region of ARHGAP24. **g** The ChIP-qPCR of CBX3 on the promoter region of ARHGAP24 in A549 and H1299 cells. Statistical significance was determined by two-side Student *t* test. Data presented as Mean ± SD with three replicates. NS not significant; **P < 0.01; ***P < 0.001. **h** The ChIP-qPCR of H3K9me3 on the promoter region of ARHGAP24 in A549 and H1299 cells. Statistical significance was determined by two-side Student *t* test. Data presented as Mean ± SD with three replicates. NS not significant; *P < 0.05; ***P < 0.001. **i** A549 cells were infected with shControl or shARHGAP24 for 48 h. Then, cells were transfected with pcDNA3.1 or Flag-CBX3 as indicated. After 24 h, cells were harvested for Western blotting analysis. Statistical significance was determined by one-way ANOVA followed by Tukey’s multiple comparisons test. For quantification of active Rac1, data presented as Mean ± SD with two replicates. NS not significant; *P < 0.05; **P < 0.01. **j**, **k** A549 cells were infected with indicated shRNAs for 72 h. Cells were collected for Western blotting analysis (**j**) and MTS assay (**k**). Statistical significance was determined by one-way ANOVA followed by Tukey’s multiple comparisons test. For quantification of active Rac1, data presented as Mean ± SD with two replicates. For MTS assay, data presented as Mean ± SD with three replicates. NS not significant; *P < 0.05; **P < 0.01; ***P < 0.001. **l**–**n** A549 cells were infected with indicated shRNAs. After 72 h puromycin selection, cells were harvested and subcutaneously injected into nude mice for xenografts assay. The image of tumor was shown in panel (**l**). The tumor growth curve was indicated in panel (**m**). The tumor mass was demonstrated in panel (**n**). Statistical significance was determined by one-way ANOVA followed by Tukey’s multiple comparisons test. Data presented as Mean ± SD with five replicates. NS not significant; ***P < 0.001.

As ARHGAP24 was reported to inhibit the Rac1 pathway [19], we next assessed whether CBX3 upregulates the levels of active Rac1 due to a reduction in the rate of deactivation via suppression of ARHGAP24 expression. CBX3 knockdown increased the protein and mRNA levels of ARHGAP24 in A549 and H1299 cells (Fig. 5a, b). Conversely, CBX3 overexpression reduced ARHGAP24 mRNA and protein levels in A549 and H1299 cells (Fig. 5c, d). Analyses using ChIP-Atlas revealed a CBX3 binding peak in the promoter of *ARHGAP24* (Fig. 5e). CBX3 represses the transcription of target genes by recognizing histone H3K9 trimethylation (H3K9me3) [20], and H3K9me3-binding sites overlap with the CBX3-binding site in the promoter region of *ARHGAP24* (Fig. 5f). ChIP-qPCR analysis revealed the presence of CBX3 and H3K9me3 in the promoter of *ARHGAP24* (Fig. 5g, h), suggesting that CBX3 regulates *ARHGAP24* expression by recognizing methylation of the *ARHGAP24* promoter. In addition, overexpression of CBX3 in ARHGAP24 ablation cells did not further increase the levels of active Rac1 compared to knockdown of ARHGAP24 alone in A549 cells (Fig. 5i). Coknockdown of ARHGAP24 and CBX3 diminished the CBX3 silencing ability to indirectly reduce Rac1 deactivation (Fig. 5J) and to inhibit the in vitro and in vivo growth of LUAD cells (Fig. 5k–n). These data suggest that CBX3 downregulates ARHGAP24 to increase the amount of active Rac1 and promote tumor growth in LUAD.

### CBX3 binds to TRIM28, TRIM24, or RBBP4 to regulate *ARHGAP24* expression in LUAD cells

**Fig. 6:**
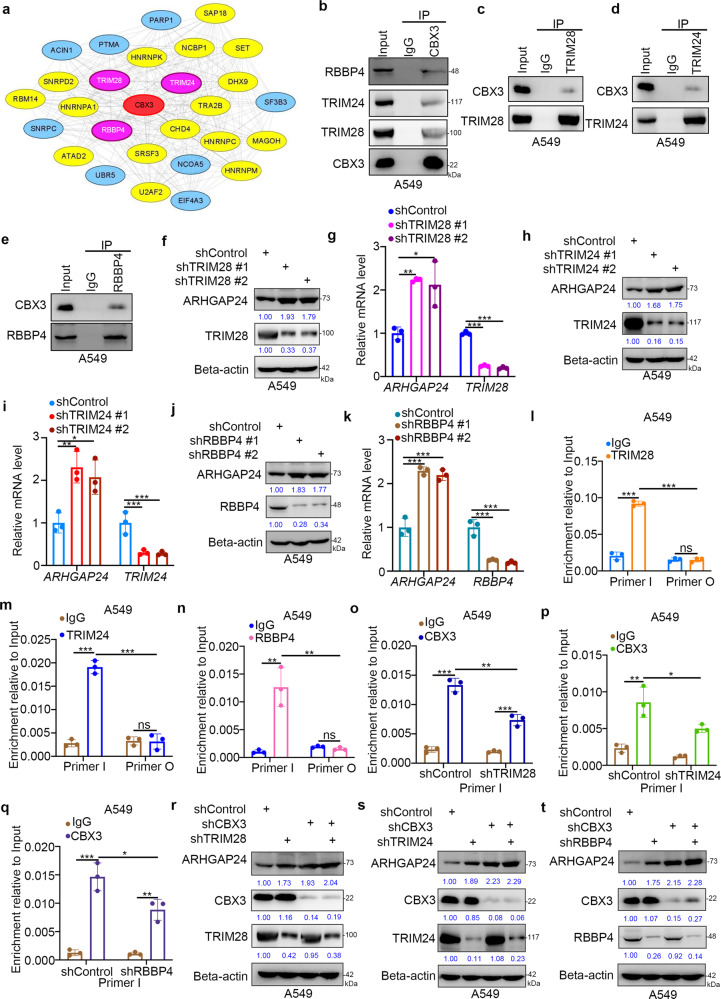
CBX3 binds to TRIM28, TRIM24, or RBBP4 to regulate ARHGAP24 expression in LUAD cells. **a** PPI network between CBX3 and proteins correlated with CBX3. **b** The whole cell lysates (WCL) of A549 were collected to undergo immunoprecipitation by using the IgG and CBX3 antibodies. Western blotting analysis was using used to detect the RBBP4, TRIM24, TRIM28 and CBX3. **c**–**e** The whole cell lysates (WCL) of A549 were collected to undergo immunoprecipitation by using the IgG and TRIM28, TRIM2, or RBBP4 antibodies, respectively. Western blotting analysis was using used to detect the RBBP4, TRIM24, TRIM28 and CBX3. **f**, **g** A549 cells were infected with shControl, shTRIM28 #1, or shTRIM28 #2 for 72 h. Cells were collected for Western blotting analysis (**f**) and RT-qPCR analysis (**g**). Statistical significance was determined by one-way ANOVA followed by Tukey’s multiple comparisons test. Data presented as Mean ± SD with three replicates. *P < 0.05; **P < 0.01; ***P < 0.001. **h**, **i** A549 cells were infected with shControl, shTRIM24 #1, or shTRIM24 #2 for 72 h. Cells were collected for Western blotting analysis (**h**) and RT-qPCR analysis (**i**). Statistical significance was determined by one-way ANOVA followed by Tukey’s multiple comparisons test. Data presented as Mean ± SD with three replicates. *P < 0.05; **P < 0.01; ***P < 0.001. **j**, **k** A549 cells were infected with shControl, shRBBP4 #1, or shRBBP4 **#**2 for 72 h. Cells were collected for Western blotting analysis (**j**) and RT-qPCR analysis (**k**). Statistical significance was determined by one-way ANOVA followed by Tukey’s multiple comparisons test. Data presented as Mean ± SD with three replicates. *P < 0.05; **P < 0.01; ***P < 0.001. **l** The ChIP-qPCR of TRIM28 on the promoter region of ARHGAP24 in A549 cells. Statistical significance was determined by two-side Student *t* test. Data presented as Mean ± SD with three replicates (*n* = 3). NS not significant; ***P < 0.001. Primer I indicated the pair of primer located in the common binding peak of RBBP4, TRIM24, TRIM28, CBX3 and H3K9me3; Primer O indicated the the pair of primer located outside the common binding peak of RBBP4, TRIM24, TRIM28, CBX3 and H3K9me3. **m** The ChIP-qPCR of TRIM24 on the promoter region of ARHGAP24 in A549 cells. Statistical significance was deter**m**ined by two-side Student *t* test. Data presented as Mean ± SD with three replicates (*n* = 3). NS not significant; ***P < 0.001. **n** The ChIP-qPCR of RBBP4 on the promoter region of ARHGAP24 in A549 cells. Statistical significance was determined by two-side Student *t* test. Data presented as Mean ± SD with three replicates (*n* = 3). NS not significant; **P < 0.01. **o** A549 cells were infected with shControl and shTRIM28 for 72 h. Cells were collected for the ChIP-qPCR of CBX3 on the promoter regi**o**n of ARHGAP24 in A549 cells. Statistical significance was determined by two-side Student *t* test. Data presented as Mean ± SD with three replicates (*n* = 3). *P < 0.05; **P < 0.01. **p** A549 cells were infected with shControl and shTRIM24 for 72 h. Cells were collected for the ChIP-qPCR of CBX3 on the promoter region of ARHGAP24 in A549 cells. Statistical significance was determined by two-side Student *t* test. Data presented as Mean ± SD with three replicates. ***P < 0.001. **q** A549 cells were infected with shControl and shRBBP4 for 72 h. Cells were collected for the ChIP-qPCR of CBX3 on the promoter region of ARHGAP24 in A549 cells. Statistical significance was determined by two-side Student *t* test. Data presented as Mean ± SD with three replicates. *P < 0.05; **P < 0.01; ***P < 0.001. **r**–**t** A549 cells were infected with indicated shRNAs for 72 h. Cells were harvested for Western blotting analysis.

Protein–protein interaction (PPI) network analyses revealed that CBX3 interacts with various proteins to regulate numerous cellular processes (Fig. 6a). Among these proteins, TRIM28, TRIM24, and RBBP4 exhibited the closest relationship with CBX3 (Fig. 6a). Intriguingly, a co-IP assay showed that CBX3 interacted with TRIM28, TRIM24, or RBBP4 in A549 and H1299 cells (Fig. 6b–e; Supplementary Fig. 4a). In addition, we performed a proximity ligation assay (PLA) to confirm the interaction between CBX3 and TRIM28, TRIM24, or RBBP4 in A549 cells (Supplementary Fig. 4b). Moreover, an immunofluorescence assay showed that CBX3 colocalized with TRIM28, TRIM24 or RBBP4 in A549 cells (Supplementary Fig. 4c). TRIM28 and TRIM24 belong to the same TRIM subfamily of proteins, which contain bromodomains [21]. We found that a TRIM28 mutant lacking the bromodomain could not bind to CBX3 in A549 cells (Supplementary Fig. 5a), suggesting that CBX3 may interact with TRIM28 through its bromodomain. As bromodomains recognize di-acetylated proteins [22, 23], we analyzed the amino acid sequence of CBX3 and identified a consensus FBP1 or TWIST1 motif for acetylation [22, 23] (Supplementary Fig. 5b). Expression of CBX3 K81R/K84R (KR) mutants mimicking deacetylated CBX3 decreased the interaction between CBX3 and TRIM28 (Supplementary Fig. 5c) and further downregulated *ARHGAP24* compared with wild-type CBX3 (Supplementary Fig. 5d–f). On the other hand, knockdown of *TRIM28*, *TRIM24*, and *RBBP4* increased ARHGAP24 expression (Fig. 6f–k; Supplementary Fig. 5g–i). Furthermore, we identified a negative correlation between the levels of ARHGAP24 and TRIM28, TRIM24, or RBBP4 in LUAD specimens (Supplementary Fig. 5j–l). Expression of the TRIM28 mutant lacking the bromodomain did not affect the expression levels of *ARHGAP24* in A549 cells (Supplementary Fig. 5m). RBBP4, together with the PRC component, catalyzes the trimethylation of histone H3 [24]. Thus, we evaluated whether CBX3 could bind TRIM28, TRIM24, and RBBP4 to regulate ARHGAP24 expression. Analysis of ChIP-seq data revealed the presence of a common binding region for TRIM28, RBBP4, TRIM24, CBX3, and H3K9me3 in the promoter of *ARHGAP24* (Supplementary Fig. 6a). ChIP-qPCR analyses confirmed the binding of TRIM28, TRIM24, and RBBP4 to the *ARHGAP24* promoter in A549 and H1299 cells (Fig. 6l–n; Supplementary Fig. 6b–d). In addition, knockdown of *TRIM28*, *TRIM24*, or *RBBP4* reduced the binding of CBX3 to the promoter of *ARHGAP24* in A549 cells (Fig. 6o–q). Conversely, overexpression of wild-type TRIM28 but not TRIM28 lacking the bromodomain enhanced CBX3 binding to the *ARHGAP24* promoter (Supplementary Fig. 6e). Furthermore, the reduction in *ARHGAP24* expression levels induced by overexpression or TRIM28 and the upregulation of ARHGAP24 expression levels induced by knockdown of TRIM28, TRIM24, or RBBP4 were not obvious after coknockdown of CBX3 in A549 cells (Fig. 6r–t; Supplementary Fig. 6f–j). In addtiton, we also found that knockdown of TRIM24 or RBBP4 combined with TRIM28 silencing could not further obviously enhance ARHGAP24 expression in A549 cells (Supplementary Fig. 6k–p), which indicated that TRIM28 is the most important factor in modulating the expression of ARHGAP24. Taken together, these data demonstrate that CBX3 forms a complex with TRIM28, TRIM24, or RBBP4 to regulate ARHGAP24 expression in LUAD.

## Discussion

Accumulating evidence shows that smoking regulates gene expression by modulating DNA methylation. For example, the methylation status of *AHRR*, *P16*, *F2RL3*, and *DAPK* has been reported as a biomarker for evaluating the progression of smoking-related diseases, such as lung cancer [25, 26]. However, unlike DNA methylation, histone methylation induced by smoking remains understudied [26]. In this study, through in silico analyses of genes involved in histone methylation, we found that the expression of the histone H3K9 methylation reader CBX3 was upregulated in current smokers with LUAD. We also identified CBX3 as a critical mediator of LUAD progression. However, the relationship between smoking and CBX3 overexpression in the lungs remains to be determined.

CBX3 is upregulated in various types of cancer, including lung cancer, osteosarcoma, liver cancer, and colorectal cancer [11, 27, 28]. However, the clinicopathological and prognostic value of CBX3 in cancer remains controversial. A previous meta-analysis demonstrated that high CBX3 expression was associated with poor prognosis in patients with lung cancer, tongue squamous cell carcinoma, digestive cancer, and urinary cancer [11]. CBX3 has been shown to interact with H3K9me3 to inhibit the expression of downstream target genes [29]. Recently, CBX3 was found to sustain the pluripotency of embryonic stem cells in an H3K26me3-dependent manner by recognizing H3K9me3 [20]. CBX3 plays a pivotal role in tumorigenesis. By inhibiting the P53/P21 pathway, CBX3 maintains the self-renewal ability of stem cells and enhances tumor growth in esophageal squamous cell carcinoma [30]. CBX3 has also been shown to regulate the c-Met/AKT/mTOR signaling pathway in glioma [31]. In prostate cancer, CBX3 has been demonstrated to regulate androgen receptor signaling and upregulate c-Myc to promote tumor progression [32, 33]. Furthermore, CBX3 has been found to be critical for the proliferation and migration of LUAD cells by inhibiting the expression of *NCOR2* and *ZBTB7A* [34]. Our bioinformatic analyses of publicly available datasets and our RNA-seq data from LUAD cells indicated that CBX3 regulates the Rho GTPase signaling pathway in LUAD cells. We also found that CBX3, in collaboration with RBBP4, TRIM28, and TRIM24, represses the expression of *ARHGAP24* to upregulate the amount of active Rac1. Nonetheless, the specific mechanisms underlying how these three proteins in complex with CBX3 regulate LUAD progression remain unknown.

Rho family GTPases belong to the Ras superfamily [35]. They have been shown to promote tumor growth and cancer cell invasion and migration and to modulate the tumor microenvironment [36–38]. Typical Rho family members include RhoA, Rac1, and Cdc42 [39]. Rho-associated protein kinases (ROCK1 and ROCK2) are important downstream effectors of Rho GTPases [40]. Rho-ROCK signaling has been extensively studied as a therapeutic target in cancer [38]. The cycle of Rho GTPases is regulated by three types of proteins: (1) guanine nucleotide exchange factors (GEFs), which activate Rho GTPases by exchanging GTP with GDP; [41] (2) GTPase-activating proteins, which promote GTP hydrolysis and inactivate Rho GTPases; [42] and (3) guanine nucleotide ionization inhibitors (GDIs), which maintain the inactive state of Rho proteins in the cytoplasm, thereby terminating the signaling pathway [43]. Rho GTPases are also regulated by posttranslational modifications, including lipid modification, phosphorylation, ubiquitination, and SUMOylation [44]. As a histone H3K9 methylation reader, CBX3 indirectly increases the amount of active Rac1 by inhibiting the GTPase-activating protein ARHGAP24. Because of the role of Rac1 in tumor progression [45], our findings provide a theoretical basis for the development of drugs targeting CBX3 to treat LUAD.

**Fig. 7:**
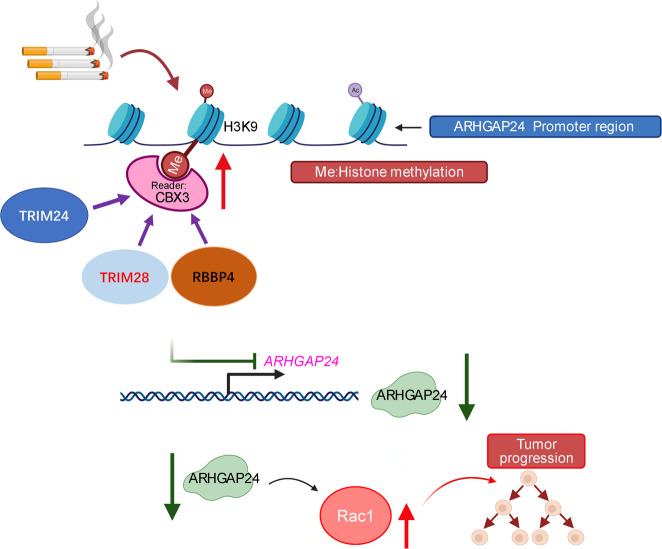
A model depicting that smoking induced CBX3 up-regulating in the lung adenocarcinoma. The overexpressed CBX3 coupled with TRIM28, TRIM24 or RBBP4 to repress ARHGAP24 and indirectly increase the amount of active Rac1, which finally promotes the progression of lung adenocarcinoma.

In conclusion, our results suggest that CBX3 is upregulated in current smokers with LUAD. CBX3 overexpression promotes LUAD cell proliferation and invasion and contributes to poor survival outcomes in patients with LUAD. This oncogenic role of CBX3 is mediated via the ARHGAP24/Rac1 pathway. Our data also suggest that TRIM28, TRIM24, and RBBP4 regulate the CBX3/ARHGAP24 axis in LUAD (Fig. 7). These findings strongly suggest that the CBX3/ARHGAP24/Rac1 axis plays a key role in smoking-induced LUAD.

## Supplementary information

Supplementary information

## Data Availability

The datasets used and/or analyzed during the current study are available from the corresponding authors (haixin.y@outlook.com) on reasonable request.
