# Supplementary material for: Smoking-associated upregulation of CBX3 suppresses ARHGAP24 expression to activate Rac1 signaling and promote tumor progression in lung adenocarcinoma
Source: Oncogene. 2021 Nov 16;41(4):538–49. doi: 10.1038/s41388-021-02114-8 (PMC8782721; doi:10.1038/s41388-021-02114-8)
Supplement: Supplementary file 1 — Supplementary information [file 41388_2021_2114_MOESM1_ESM.pdf]

Smoking-associated upregulation of CBX3 suppresses ARHGAP24 expression to activate Rac1 signaling and promote tumor progression in lung adenocarcinoma

Xin Jin, Bin Zhang, Hao Zhang, Haixin Yu

Supplementary figure 1

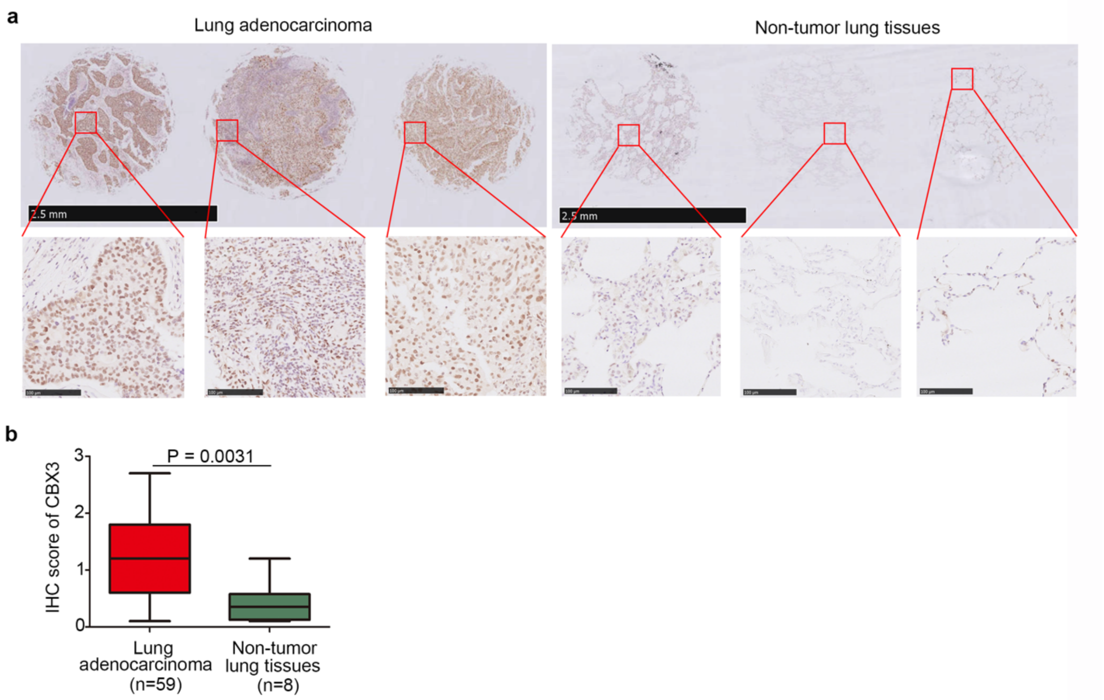

Supplementary figure 1. a-b, the tissue microarray of lung adenocarcinoma was stained with CBX3 (lung adenocarcinoma tissues n = 59, non-tumor lung tissues n = 8). The typical IHC images stained with CBX3 were shown in panel a. The size of the scale bar on microscopy images as indicated in the figure. P values as indicated.

Supplementary figure 2

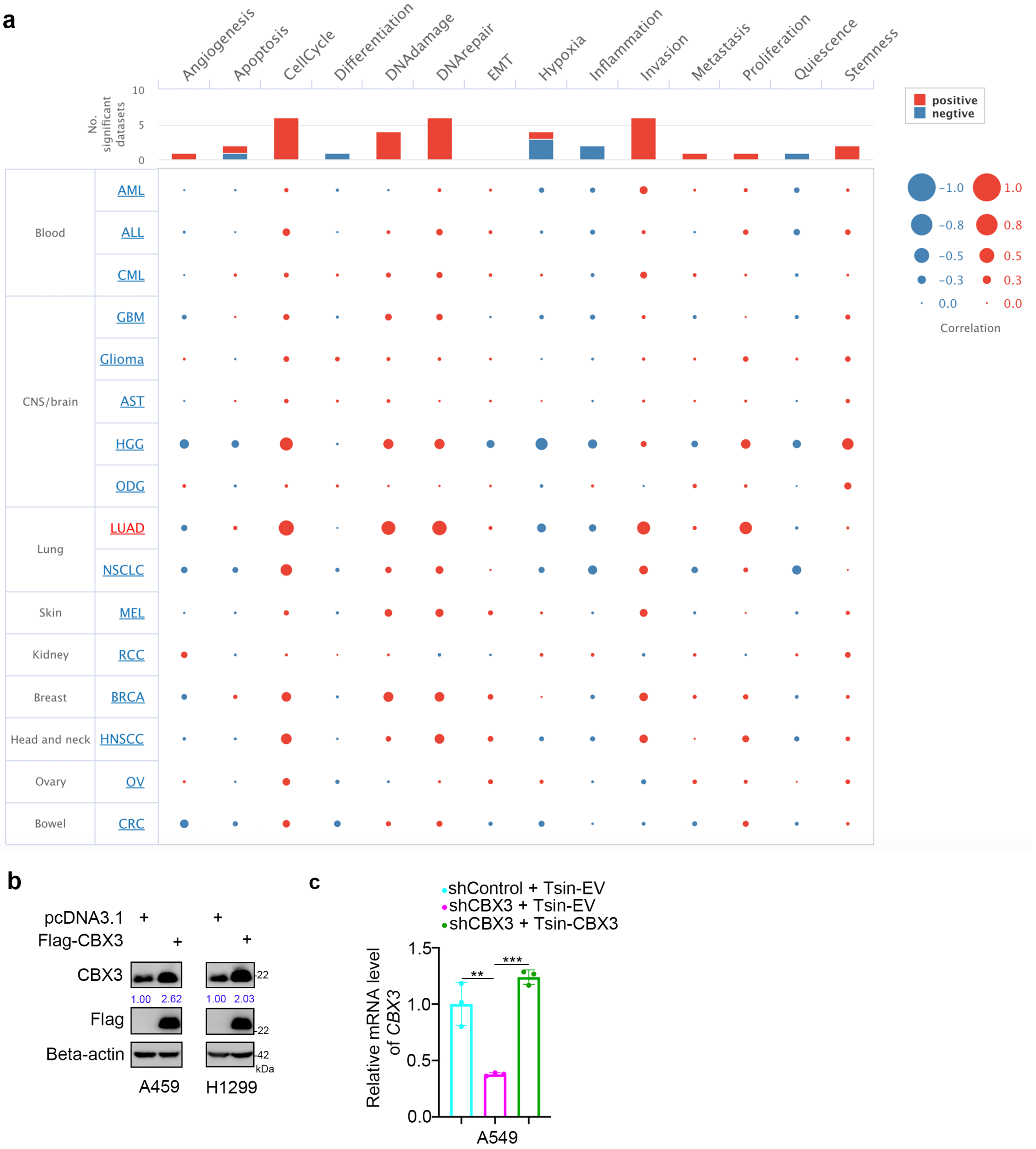

Supplementary figure 2. a, The single cells seq analysis of various types of cancer. **b**, A549 and H1299 cells were transfected with pcDNA3.1 or Flag-CBX3 for 24 h. Cells were collected for Western blot analysis. **c**, A549 cells were infected withTsin-EV+shControl, Tsin-EV+shCBX3, or Tsin-CBX3+shCBX3 as indicated for 72 h. Cells were harvested for RT-qPCR analysis. Statistical significance was determined by one-way ANOVA followed by Tukey's multiple comparisons test.

Data presented as Mean ± SD with three replicates (n = 3). **, P < 0.01; ***, P < 0.001.

Supplementary figure 3

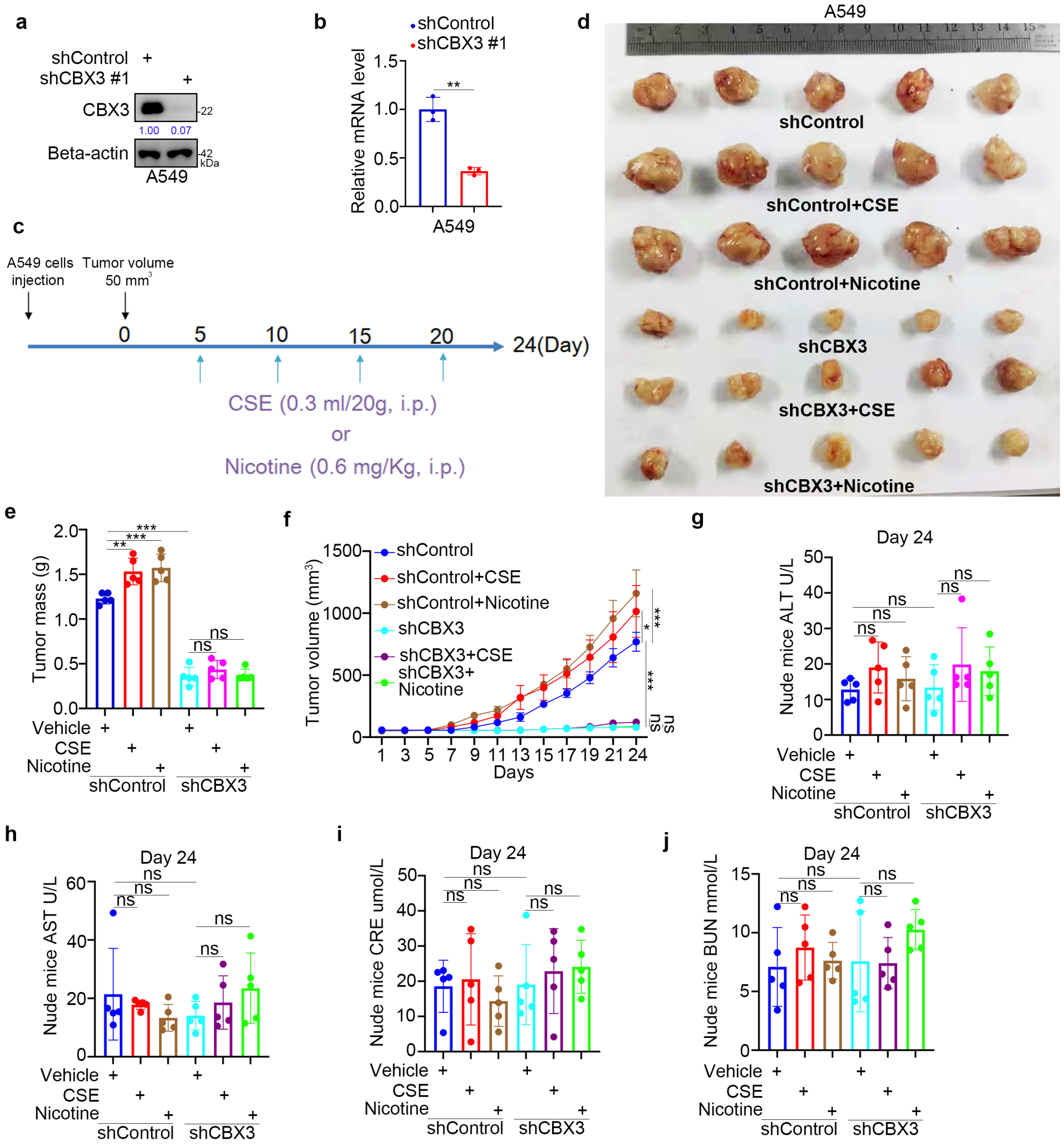

Supplementary figure 3. a-f, A549 cells were infected with shControl or shCBX3 #1. After 72 h puromycin selection, cells were harvested for Western blotting analysis (a) or RT-qPCR analysis (b). For RT-qPCR analysis, data presented as Mean ± SD with three replicates (n = 3). **, P < 0.001. Statistical significance was determined by

paired student t-test. After confirmed that CBX3 was silenced in the A549 cells, the shControl and shCBX3 #1 group cells were subcutaneously injected into the nude mice randomly. Then mice were treated with or without vehicle, CSE (0.3 ml/20g, i.p.), or Nicotine (0.6mg/Kg, i.p.) as indicated in panel c. The image of tumor was shown in panel d. The tumor mass was demonstrated in panel e. The tumor growth curve was indicated in panel f. Statistical significance was determined by one-way ANOVA followed by Tukey's multiple comparisons test. Data presented as Mean ± SD with five replicates. Ns, not significant; *, $P < 0.05$; ***, $P < 0.001$. **g-j**, On day 24, after harvested xenografts from nude mice, we detected the aminoleucine transferase (ALT) (g), aspartate transaminase (AST) (h), creatinine (CRE) (i), blood urea nitrogen (BUN) (j) of each group of nude mice. Statistical significance was determined by one-way ANOVA followed by Tukey's multiple comparisons test. Data presented as Mean ± SD with five replicates. Ns, not significant.

Supplementary figure 4

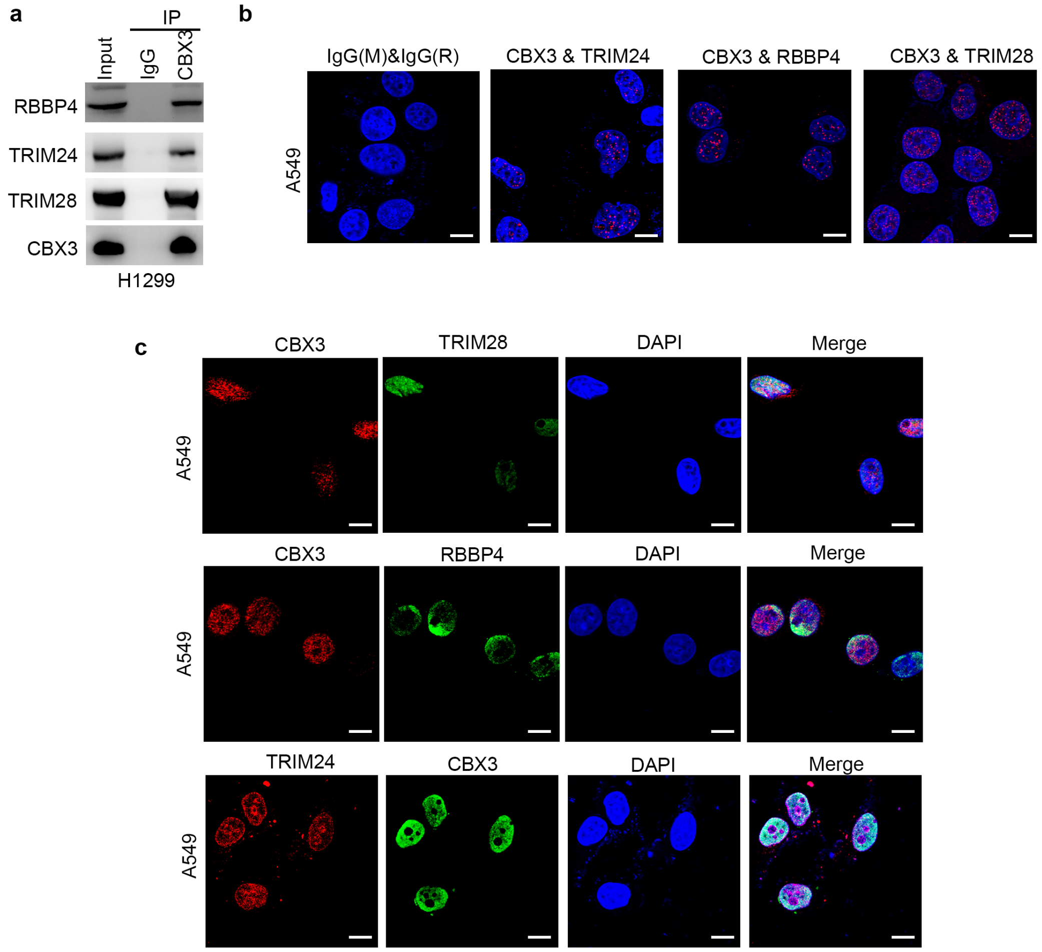

Supplementary figure 4. a, The whole cell lysates (WCL) of H1299 were collected to undergo immunoprecipitation by using the IgG or CBX3 antibodies, respectively. Western blotting analysis was using used to detect the RBBP4, TRIM24, TRIM28 and CBX3.**b**, the PLA by using the IgG(Moues) & IgG(Rabbit), CBX3 & TRIM24, CBX3 & RBBP4, or CBX3 & TRIM28 antibodies to verify the interaction between CBX3 and TRIM24, RBBP4 or TRIM28 in A549 cells. The scale bar on microscopy images as indicated. **c**, A459 cells were stained with CBX3 and TRIM28, CBX3 and TRIM24, CBX3 and RBBP4 antibodies. These cells were analyzed by immunofluorescence assay. The scale bar on microscopy images as indicated.

Supplementary figure 5

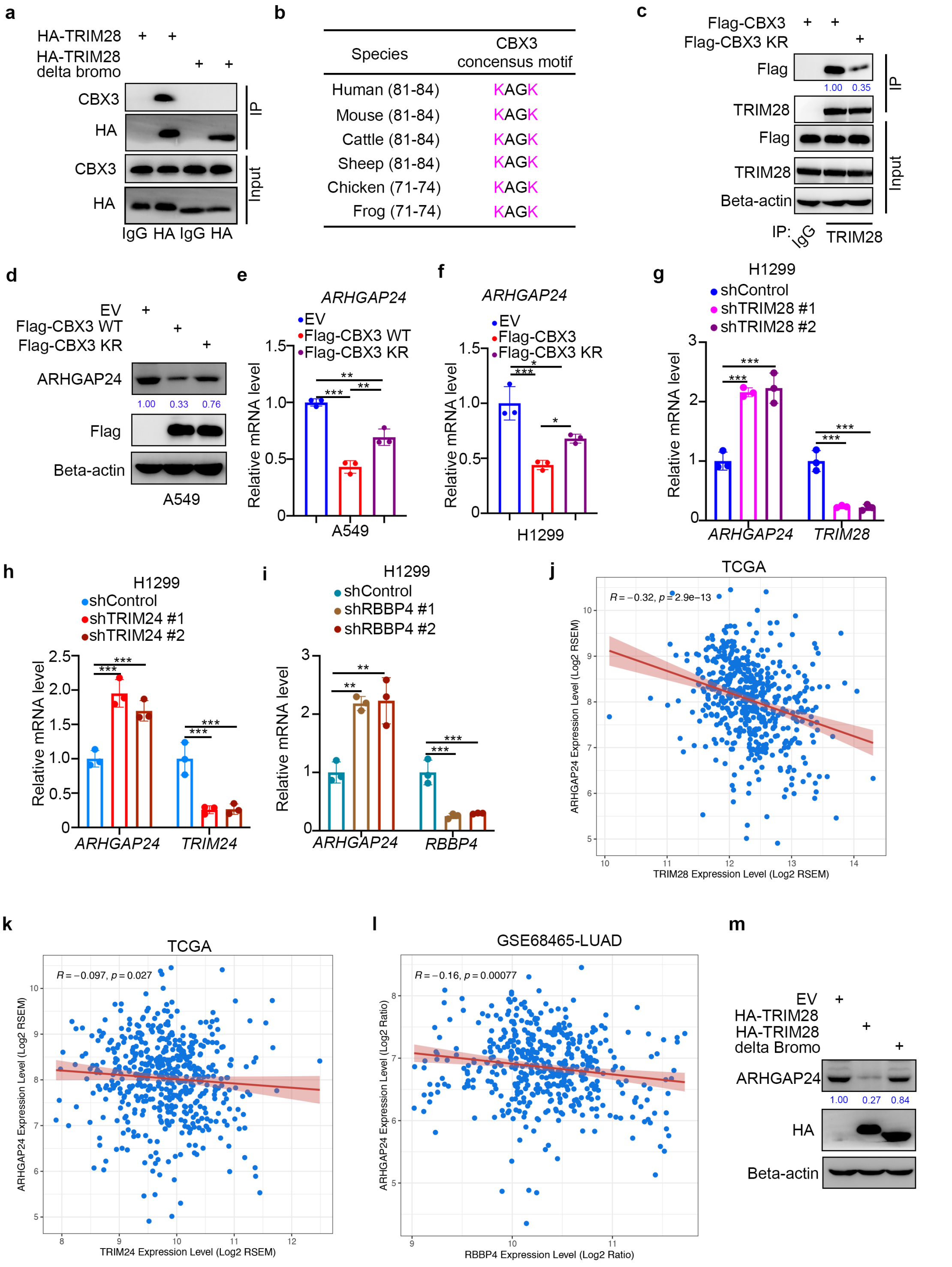

Supplementary Figure 5. a, A549 cells were transfected with indicated plasmids for 24 h. Cells were harvested for Western blotting analysis. **b,** a schematic diagram depicting that the "KAGK " consensus motif of CBX3. **c,** A549 cells were transfected

with indicated plasmids for 24 h. Cells were harvested for Western blotting analysis. **d-f**, A549 and H1299 cells were transfected with indicated plasmids for 24 h. Cells were harvested for Western blotting analysis (d) and RT-qPCR analysis (e and f). Data presented as Mean± SD (n = 3). *, $P < 0.05$; **, $P < 0.01$; ***, $P < 0.001$. **g-i**, H1299 cells were infected with indicated shRNAs for 72 h. Cells were harvested for RT-qPCR analysis. Data presented as Mean± SD (n = 3). *, $P < 0.05$; **, $P < 0.01$; ***, $P < 0.001$. **j-l**, analysis the correlation between TRIM28, TRIM24 or RBBP4 and ARHGAP24 in lung adenocarcinoma in indicated datasets. **m**, A549 cells were transfected with indicated plasmids for 24 h. Cells were harvested for Western blotting analysis.

Supplementary figure 6

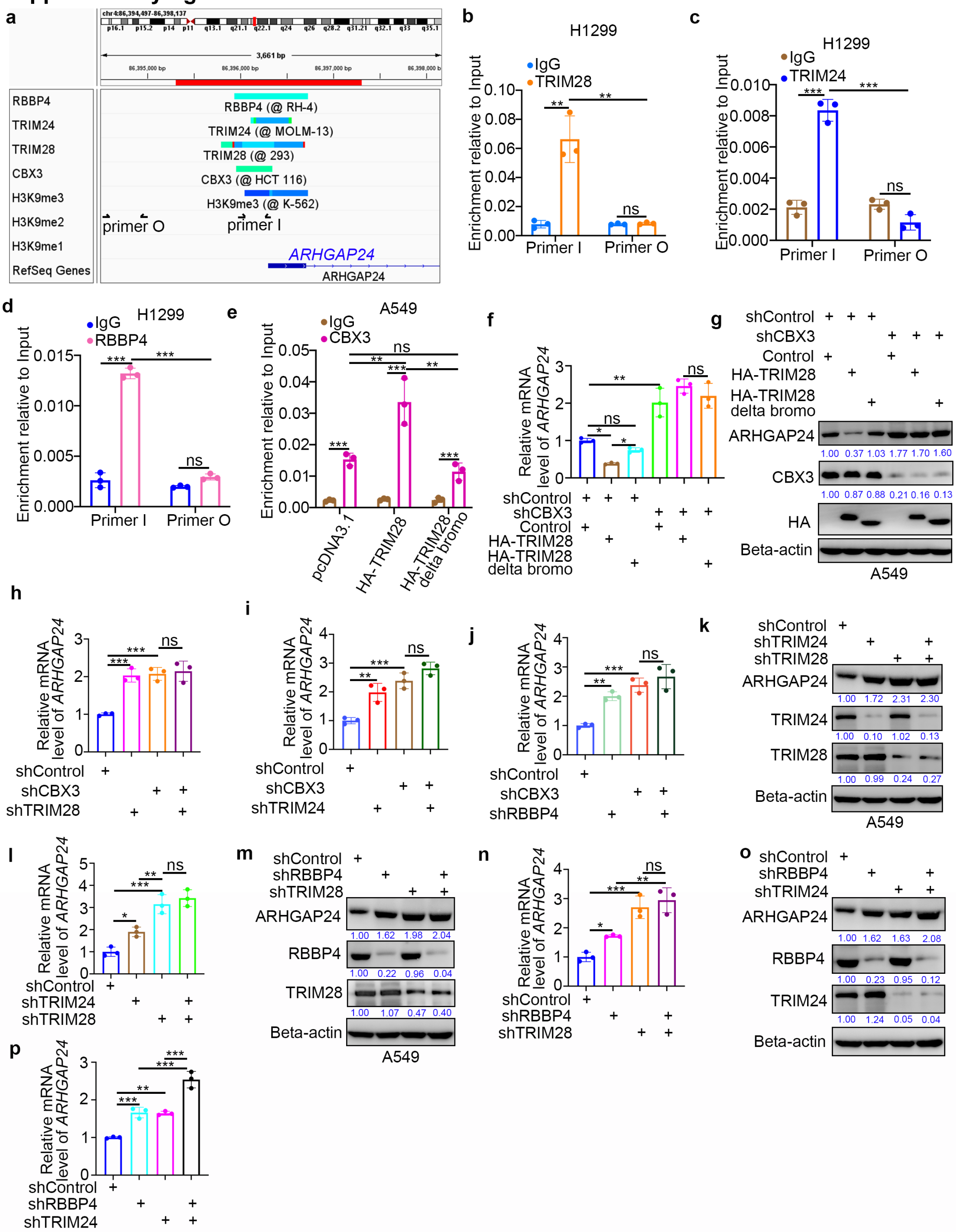

Supplementary Figure 6. a, the ChIP-seq of CBX3, TRIM28, TRIM24, RBBP4 and H3K9me3 on the promoter region of ARHGAP24. Primer I indicated the pair of primer located in the common binding peak of RBBP4, TRIM24, TRIM28, CBX3 and H3K9me3; Primer O indicated the pair of primer located outside the common binding

peak of RBBP4, TRIM24, TRIM28, CBX3 and H3K9me3 as indicated.**b**, the ChIP-qPCR of TRIM28 on the promoter region of ARHGAP24 in H1299 cells. Data presented as Mean ± SD with three replicates (n = 3). Ns, not significant; **, P < 0.01. **c**, the ChIP-qPCR of TRIM24 on the promoter region of ARHGAP24 in H1299 cells. Data presented as Mean ± SD with three replicates (n = 3). Ns, not significant; **, P < 0.01; ***, P < 0.001. **d**, the ChIP-qPCR of TRIM24 on the promoter region of ARHGAP24 in H1299 cells. Data presented as Mean ± SD with three replicates (n = 3). Ns, not significant; ***, P < 0.001. **e**, A549 cells were transfected with indicated plasmids for 24 h. Cells were collected for the ChIP-qPCR of CBX3 on the promoter region of ARHGAP24 in A549 cells. Data presented as Mean ± SD with three replicates (n = 3). Ns, not significant; **, P < 0.01; ***, P < 0.001. **f and g**, A549 cells were infected with shControl or shCBX3 for 48 h. Then, cells were transfected with indicated plasmids for another 24 h. Cells were collected for RT-qPCR analysis (f) and Western blotting analysis (g). Data presented as Mean ± SD with three replicates (n = 3). Ns, not significant; *, P < 0.05; **, P < 0.01. **h-j**, A549 cells were infected with indicated shRNAs for 72 h. Cells were harvested for RT-qPCR analysis. Data presented as Mean ± SD with three replicates (n = 3). Ns, not significant; **, P < 0.01; ***, P < 0.001. **k and l**, A549 cells were infected with indicated shRNAs for 72 h. Cells were collected for Western blotting analysis (k) and RT-qPCR analysis (l). Data presented as Mean ± SD with three replicates (n = 3). Ns, not significant; *, P < 0.05; **, P < 0.01; ***, P < 0.001. **m and n**, A549 cells were infected with indicated shRNAs for 72 h. Cells were collected for Western blotting analysis (m) and

RT-qPCR analysis (n). Data presented as Mean ± SD with three replicates (n = 3). Ns, not significant; *, P < 0.05; **, P < 0.01; ***, P < 0.001. **o and p**, A549 cells were infected with indicated shRNAs for 72 h. Cells were collected for Western blotting analysis (o) and RT-qPCR analysis (p). Data presented as Mean ± SD with three replicates (n = 3). **, P < 0.01; ***, P < 0.001.

Supplementary Material and Methods

Public datasets for data mining and bioinformatics analysis

1）TCGA-LUAD：Transcriptome data and clinical information of LUAD patients were obtained from the GDC data portal (https://portal.gdc.cancer.gov/). Data from 522 LUAD patients and 60488 genes were acquired. Of these patients, 59 samples had matched normal tissues.

2）TCGA-LUSC：Transcriptome data and clinical information of LUSC patients were obtained from the GDC data portal (https://portal.gdc.cancer.gov/). Data from 504 LUSC patients and 60488 genes were acquired. Of these patients, 49 samples had matched normal tissues.

3）GSE68465: Transcriptome data and clinical information of LUAD patients were obtained from the GEO database (https://www.ncbi.nlm.nih.gov/). Data from 443 LUAD patients and 22283 genes were acquired. Of these patients, 19 samples had matched normal tissues.

4）CPTAC-LUAD: Proteomics data and clinical information of LUAD patients were obtained from the CPTAC data portal (https://proteomics.cancer.gov/programs/cptac). Data from 113 LUAD patients and 11029 proteins were acquired. Of these patients, 102 samples had matched normal tissues.

5）EXP0026: Transcriptome data of single cells from LC-2/ad cell line were obtained from the CancerSEA database (http://biocc.hrbmu.edu.cn/CancerSEA/). Data from 245 single cells and 14206 coding genes were acquired.

6）EXP0027: Transcriptome data of single cells from A549 cell line were obtained from the CancerSEA database (http://biocc.hrbmu.edu.cn/CancerSEA/). Data from 218 single cells and 11520 coding genes were acquired.

Identification of key genes involved in the progression of LUAD

Univariate cox regression analysis was first applied to identify genes associated with the RFS of LUAD patients in TCGA-LUAD dataset. Then, genes with $P < 0.05$ went through lasso-cox regression analysis with 10-fold cross-validation to further select genes associated with the RFS of LUAD. The lasso selection process was replicated by 1000 times.

Identification of smoking-related genes

Since nonsmokers are underrepresented in lung cancer, smoking-related genes were mainly identified between current smokers and former smokers in this study. We also required that the smoking-related genes were differentially expressed in both the TCGA-LUAD and TCGA-LUSC datasets.

Survival analysis

LUAD patients were divided into two groups according to the median expression level of the key gene. The differences in RFS and OS between the high and low expression groups were evaluated by the Kaplan–Meier method, followed by a log-rank test.

GSEA for the key gene

LUAD patients were first divided into two groups according to the median expression level of the key gene. Then, differential expression analysis was applied between the high and low expression groups. Input genes for GSEA were sorted by their logFC values. Signaling pathways activated or suppressed by the key gene were decided by the NES value derived from GSEA.

Downstream targets of the key gene

Downstream targets of the key gene were determined by ChIP-seq and correlation analysis. Binding site in the targeted gene promoter of the key protein or methylation were obtained from ChIP-Atlas database (https://chip-atlas.org/). Correlation analysis between key gene/protein and downstream targeted genes was applied to further validate the ChIP-seq results ($|r| \geq 0.30$ & $P < 0.05$).

PPI network between key protein and correlated proteins

Correlation analysis was applied to identify proteins correlated with the key protein ($|r| \geq 0.30$ & $P < 0.05$). PPI network among key protein and correlated proteins was constructed using STRING v11.0 (https://string-db.org/).

Statistical analysis and visualization

Microsoft R Open v4.0.2 was used for data mining, bioinformatics analysis and visualization in transcriptomics and proteomics data. IGV v2.9.0 was used for analysis and visualization of ChIP-seq data. STRING v11.0 was used for analysis and visualization of PPI network.

Cell proliferation assay

Cell proliferation was determined using an MTS assay. Lung adenocarcinoma cells (1×10^4 cells/well) were seeded in 96-well plates and incubated for 24h. Then, the medium was replaced with fresh medium containing MTS reagent (Cat. No. ab197010, Abcam), and the absorbance of each well at 490 nm was determined the cell growth ability.

For colony formation assay, lung adenocarcinoma cells (500 cells/well) were cultivated into 6-well plates and incubated in RPMI-1640 medium with 10% FBS at 37°C for 2 weeks. Then the cells were fixed in methanol for 30 min and stained with 1% Crystal Violet Staining Solution for 30 mins and then washed with PBS 3 times. Finally, the number of colonies was calculated.

Cell invasion assay

The in vitro cell invasion assay was applied by using a Bio-Coat Matrigel invasion chamber (BD Biosciences). Each transwell chamber was coated with 50 μL matrigel (1: 8, CORNING). Cells with 100 μL serum-free RPMI-1640 medium were incubated in the upper chamber for 24 h (lower chamber containing complete medium). Then cells were fixed in methanol for 15 min and then stained with 1% crystal violet for 20

min. Cell images were taken in three fields under the microscope, and the number of cells penetrating the membrane was counted.

Xenografts assay

Ethical approval was obtained by the Ethics Committee of Tongji Medical College, Huazhong University of Science and Technology for all animal procedures. BALB/c-nude mice (4-5 weeks old, 18-20 g) were obtained from Vitalriver (Beijing, China). A459 cells were transduced with different lentiviral particles. After puromycin selection for 72 h, cells (1×10^7 per mouse) were subcutaneously injected into the back of mice. The procedure of xenografts assay was described previously [1]. At the study endpoint, the volume and mass of xenografts were measured. Nicotine (Cat No. MED24104) were purchased from Medbio (CN).

RNA interference and plasmids transfection

Lipofectamine 2000 (Thermo Fisher Scientific, CN) was applied for plasmids transfections following the manufacture's protocol. Flag-CBX3 was cloned into the CMV-MCS-3xFlag-SV40-neomycin vector by GENECHEM (Shanghai, CN). The lentivirus-based control and gene-specific shRNAs, obtained from Sigma-Aldrich, combined with pVSV-G were employed to produce different lentiviral particles in 293T cells through lipofectamine 2000. 24 h post-transfection, the cell culture medium was replaced with fresh RPMI-1640 medium. After 48 h, the cell culture medium containing the viral particles was harvested and added into the culture

medium of lung adenocarcinoma cell lines.

Preparation of CSE

Commercial cigarettes (Hongta, China, 1.0 mg nicotine per cigarette; 10 mg tar per cigarette) were smoked using a vacuum pump for 5 min each, and smoke per 10 mL of serum-free cell growth medium was used to generate 100% CSE-PBS solution. The CSE was prepared 30 min before use, and the pH was regulated to 7.4. The solution was sterilized by filtration with 0.22 μm of filter membrane [2].

Proximity ligation assay (PLA)

The A549 cells were fixed by the blocking solution following the manufacture's protocol (Duolink in situ-fluorescence, sigma). Then, the primary antibodies CBX3 11650-2-AP, proteintech; 1:100 dilution) and TRIM28 (66630-1-Ig, proteintech; 1:200 dilution), TRIM24 (66324-1-Ig, proteintech; 1:200 dilution), or RBBP4 (66060-1-Ig, proteintech; 1:400 dilution), IgG (Rabbit) (3900, Cell Signaling Technology; 1:5000 dilution) and IgG (Mouse) (53484, Cell Signaling Technology; 1:5000 dilution) were applied to incubated with the cells for 2 h at 37ºC. Then, cells were washed with 1 X wash buffer and incubated with PLA probe for 1 h at 37ºC. The Ligation-Ligase was added to cells at 37ºC. 30 mins later, cells were incubated with Amplification-Polymerase solution for 100 mins. The Duolink In Situ Mounting Medium with DAPI was added to cells to take photos under confocal microscope.

Immunofluorescence assay (IFA)

IFA was performed as previously described [3]. Briefly, cells were fixed in 4% paraformaldehyde for 15 min. After washed in 1 X PBS for three times, fixed cells were permeabilized with 0.2% Triton X-100 for 20 min, washed in 1 X PBS and then blocked in PBS supplemented with 10% goat serum. Cells were incubated with indicated primary antibody at overnight. After washed three times with 1X PBS, cells were incubated with secondary antibody that was conjugated with Alexa Fluor 488 dye (Cat No. srbAF488-1 or Cat No. sms1AF488-1, proteintech) or Alexa Fluor 594 dye (Thermo Fisher Scientific) for 1 hr at room temperature. After washed three times with 1X PBS, cells were counterstained with Vectashield (Vector Laboratories) containing DAPI (4', 6-diamidino-2-phenylindole). Images were captured using confocal microscope.

ALT, AST, CRE and BUN detection assay

The blood serum of nude mice was collected. The ALT, AST, CRE or BUN were tested following the manufacture's protocol of Alanine aminotransferase Assay Kit (Cat No. C009-2-1, Nanjing jiancheng bioengineering institute, China), Aspartate aminotransferase Assay Kit (Cat No. C010-2-1, Nanjing jiancheng bioengineering institute, China), CREatinine Assay Kit (Cat No. C011-1-1, Nanjing jiancheng bioengineering institute, China) and Urea Assay Kit (Cat No. C013-2-1, Nanjing jiancheng bioengineering institute, China).

Reference

1 Zhang B, Cheng X, Zhan S, Jin X, Liu T. MIB1 upregulates IQGAP1 and promotes pancreatic cancer progression by inducing ST7 degradation. *Mol Oncol* 2021.

2 Liu W, Tan X, Shu L, Sun H, Song J, Jin P *et al*. Ursolic acid inhibits cigarette smoke extract-induced human bronchial epithelial cell injury and prevents development of lung cancer. *Molecules* 2012; 17: 9104-9115.

3 Jin X, Ding D, Yan Y, Li H, Wang B, Ma L *et al*. Phosphorylated RB Promotes Cancer Immunity by Inhibiting NF-kappaB Activation and PD-L1 Expression. *Mol Cell* 2019; 73: 22-35 e26.

Table S1: Sequences of RT-qPCR primers

Species	Gene	Forward (5'-3')	Reverse (5'-3')
Human	*GAPDH*	CCAGAACATCATCCCTGCCT	CCTGCTTCACCACCTTCTTG
Human	*CBX3*	GAGATGCTGCTGACAAACCA	TATTTGCCTCTTTCGCCAGC
Human	*ARHGAP24*	ACAAGTCTGAGTCACCCCAG	GGGGTGGTTTGGGTTTTCTC
Human	*TRIM24*	ATCCCCAGTGACCAACAACA	AGGCTGATCTGTGTTGGGAA
Human	*TRIM28*	AGTGGATGTCAAGATGGCCA	TGGTGCTTCTGGATCTTGGT
Human	*RBBP4*	AGGAGAAGTAAACAGGGCCC	CCTTCTGATGTCCACGGAGA

Table S2: Sequences of ChIP-qPCR primers

Species	Gene	Forward (5'-3')	Reverse (5'-3')
Human	*ARHGAP24* *(Primer I)*	TGCACTTGAAAGGAACGA GC	TGGTAGCTTCACAGGACTCG
Human	*ARHGAP24* *(Primer O)*	CCCAGTAACCACCCTGAGT T	TGGACTCAAAAGCCTCAGGA

Table S3: Sequences of gene-specific shRNAs

shCBX3-1	5′-CCGGCTGGCGAAAGAGGCAAATATGCTCGAGCATATTTGCCTCTTTCGCCAGTTTTTG-3′
shCBX3-2	5′- CCGGCGACGTGTAGTGAATGGGAAACTCGAGTTTCCCATTCACTACACGTCGTTTTT-3′
shARHGAP24	5′- CCGGCCTGGATGTACAGAAGTCTAACTCGAGTTAGACTTCTGTACATCCAGGTTTTTTG-3′
shTRIM28-1	5′- CCGGCCTGGCTCTGTTCTCTGTCCTCTCGAGAGGACAGAGAACAGAGCCAGGTTTTT-3′
shTRIM28-2	5′- CCGGCTGAGACCAAACCTGTGCTTACTCGAGTAAGCACAGGTTTGGTCTCAGTTTTT-3′

shTRIM24-1	5′- CCGGCCATGAAATGAGCCTGGCTTTCTCGAGAAAGCCAGGCTCATTTCATGGTTTTT-3′
shTRIM24-2	5′-CCGGCCTTGTTAAGTTAACACCTATCTCGAGATAGGTGTTAACTTAACAAGGTTTTTG-3′
shRBBP4-1	5′- CCGGGCCTTTCTTTCAATCCTTATACTCGAGTATAAGGATTGAAAGAAAGGCTTTTTG-3′
shRBBP4-2	5′- CCGGTGGTCATACTGCCAAGATATCCTCGAGGATATCTTGGCAGTATGACCATTTTTG-3′

Table S4: Clinical data summary for lung cancer patients from TCGA

Characteristics	TCGA-LUAD (N=522)		TCGA-LUSC (N=504)	
	n	%	n	%
Age (year)				
⩾65	280	53.6	325	64.5
<65	223	42.7	170	33.7
NA	19	3.6	9	1.8
Gender				
Female	280	53.6	131	26.0
Male	242	46.4	373	74.0
Smoking status				
Never smoker	75	14.4	18	3.6
Former smoker	311	59.6	340	67.5
Current smoker	122	23.4	134	26.6
NA	14	2.7	12	2.4
TNM stage				
Stage I	279	53.4	245	48.6
Stage II	124	23.8	163	32.3
Stage III	85	16.3	85	16.9
Stage IV	26	5.0	7	1.4
NA	8	1.5	4	0.8
Race				
White	393	75.3	351	69.6
Black or African American	53	10.2	31	6.2
Asian	8	1.5	9	1.8
American Indian or Alaska Native	1	0.2	0	
NA	67	12.8	113	22.4
Survival				
Alive	329	63.0	285	56.5
Dead	184	35.2	213	42.3
NA	9	1.7	6	1.2

NA, not available

Table S5: Clinical data summary for LUAD samples used for IHC

Characteristics	IHC dataset (N=59)	
	n	%
Age		
<45	5	8.5
45 to 60	29	49.2
60 to 75	23	39.0
⩾75	2	3.4
Gender		
Female	23	39.0
Male	36	61.0
Tumor		
T1	2	3.4
T2	52	88.1
T3	4	6.8
T4	1	1.7
Node		
N0	38	64.4
N1	18	30.5
N2	3	5.1
N3	0	0.0
Metastasis		
M0	58	98.3
M1	1	1.7
Stage		
I	32	54.2
II	19	32.2
III	7	11.9
IV	1	1.7
Grade		
1	2	3.4
2	34	57.6
2 to 3	1	1.7
4	22	37.3
